# Right Ventricular Adaptation Is Associated with the Glu298Asp Variant of the NOS3 Gene in Elite Athletes

**DOI:** 10.1371/journal.pone.0141680

**Published:** 2015-10-30

**Authors:** Zsolt Szelid, Árpád Lux, Márton Kolossváry, Attila Tóth, Hajnalka Vágó, Zsuzsanna Lendvai, Loretta Kiss, Pál Maurovich-Horvat, Zsolt Bagyura, Béla Merkely

**Affiliations:** 1 Semmelweis University Heart and Vascular Center, Budapest, Hungary; 2 MTA-SE Cardiovascular Imaging Research Group, Heart and Vascular Center, Semmelweis University, Budapest, Hungary; Victoria University, AUSTRALIA

## Abstract

Nitric oxide (NO), an important endogenous pulmonary vasodilator is synthetized by the endothelial NO synthase (*NOS3*). Reduced NO bioavailability and thus the Glu298Asp polymorphism of *NOS3* may enhance right ventricular (RV) afterload and hypertrophic remodeling and influence athletic performance. To test this hypothesis world class level athletes (water polo players, kayakers, canoeists, rowers, swimmers, n = 126) with a VO_2_ maximum greater than 50ml/kg/min were compared with non-athletic volunteers (n = 155). Cardiopulmonary exercise tests and cardiac magnetic resonance imaging (cMRI) were performed to determine structural or functional changes. Genotype distribution of the *NOS3* Glu298Asp polymorphism was not affected by gender or physical performance. Cardiac MRI showed increased stroke volume with eccentric hypertrophy in all athletes regardless of their genotype. However, the Asp allelic variant carriers had increased RV mass index (32±6g versus 27±6g, *p*<0.01) and larger RV stroke volume index (71±10ml versus 64±10ml, *p*<0.01) than athletes with a Glu/Glu genotype. Genotype was not significantly associated with athletic performance. In the non-athletic group no genotype related differences were detected. The association between the *NOS3* Glu298Asp polymorphism and RV structure and dimension in elite athletes emphasizes the importance of *NOS3* gene function and NO bioavailability in sport related cardiac adaptation.

## Introduction

Intensive physical conditioning results in cardiac adaptive responses known as the “athlete’s heart.” These changes include development of left- and right ventricular (LV, RV respectively) hypertrophy and increased stroke volumes, which correlate with maximal work capacity and are known to be load and sports discipline dependent [1–4].

Recent recognition of the endurance training-induced disproportionate right ventricular load highlighted a more pronounced fatigue and a potentially larger damage of the right—compared to the left—heart chamber [5,6]. Diminished right ventricular contractile reserve and limited pulmonary vascular response put an almost ten-fold stress on the RV wall during exercise [5]. Therefore, chronic RV overload evokes structural, electrical and functional changes, which may result in the development of exercise-induced arrhythmogenic RV cardiomyopathy and increase the incidence of ventricular arrhythmias [6,7].

Nitric oxide (NO) is an important cardiovascular regulator, modulating flow patterns in the pulmonary circulation and influencing exercise performance through effects on skeletal muscles and cardiac function [8–11]. Endothelial NO synthase is the main source of NO in the heart and its genetic variants may have an impact on NO bioavailability.

Examination of several genetic variants of the *NOS3* gene in different clinical conditions displayed significant ethnicity- and genotype distribution-related differences. The three most widely studied *NOS3* polymorphisms include the G894CT (Glu298Asp) in exon 7, the 4a/5b 27-basepair variable number of tandem repeats in intron 4, and the T-786C variant in the promoter region [12].

The 894 G/T variant of the *NOS3* gene predicts a Glu to Asp amino acid substitution at codon 298 in the mature protein, which increases NOS3 susceptibility to enzymatic cleavage *in vitro* and may result in decreased NO production [13]. Aspartate homozygosity was linked with carotid and coronary artery atherosclerosis, hypertension and an increased risk to develop ischemic heart disease or dilated cardiomyopathy (DCM) in patients [14–19]. These data however were not supported by the Human Genome Epidemiology (HuGE) review, probably due to the strong interaction between ethnic and environmental factors and phenotypes [20].

The *NOS3* Glu298Asp polymorphism has already been associated with exercise induced cardiac phenotypes. Carriers of the Asp allelic variant developed higher stroke volumes and lower heart rates during sub-maximal exercise in endurance trained women [21]. In triathletes even lower finishing times and better athletic performance were attributed to the *NOS3* 298 Glu/Glu genotype [22]. Despite these reports genotype distribution of this variant shows no difference between elite endurance athletes and sedentary controls [23].

These associations of the *NOS3* 298 polymorphism and cardiac function—with special emphasis on athletic adaptation—may suggest changes in physiologic or even pathologic cardiac remodeling and to our knowledge these associations were not yet studied.

We hypothesized that altered *NOS3* function and endogenous NO production may influence myocardial hypertrophy in athletes. Therefore, *NOS3* 298 genotype (dbSNP: rs1799983, OMIM: +163729) was determined and athletic cardiac adaptation using cardiac magnetic resonance imaging (cMRI) was investigated in a selected population of world class athletes. Within the framework of a national screening program for the Hungarian national sport teams the most elite representatives of athletic disciplines with substantial endurance training incorporating power components were selected and compared with non-athlete individuals.

## Materials and Methods

### Selection of candidate individuals and study protocol

**Fig 1:**
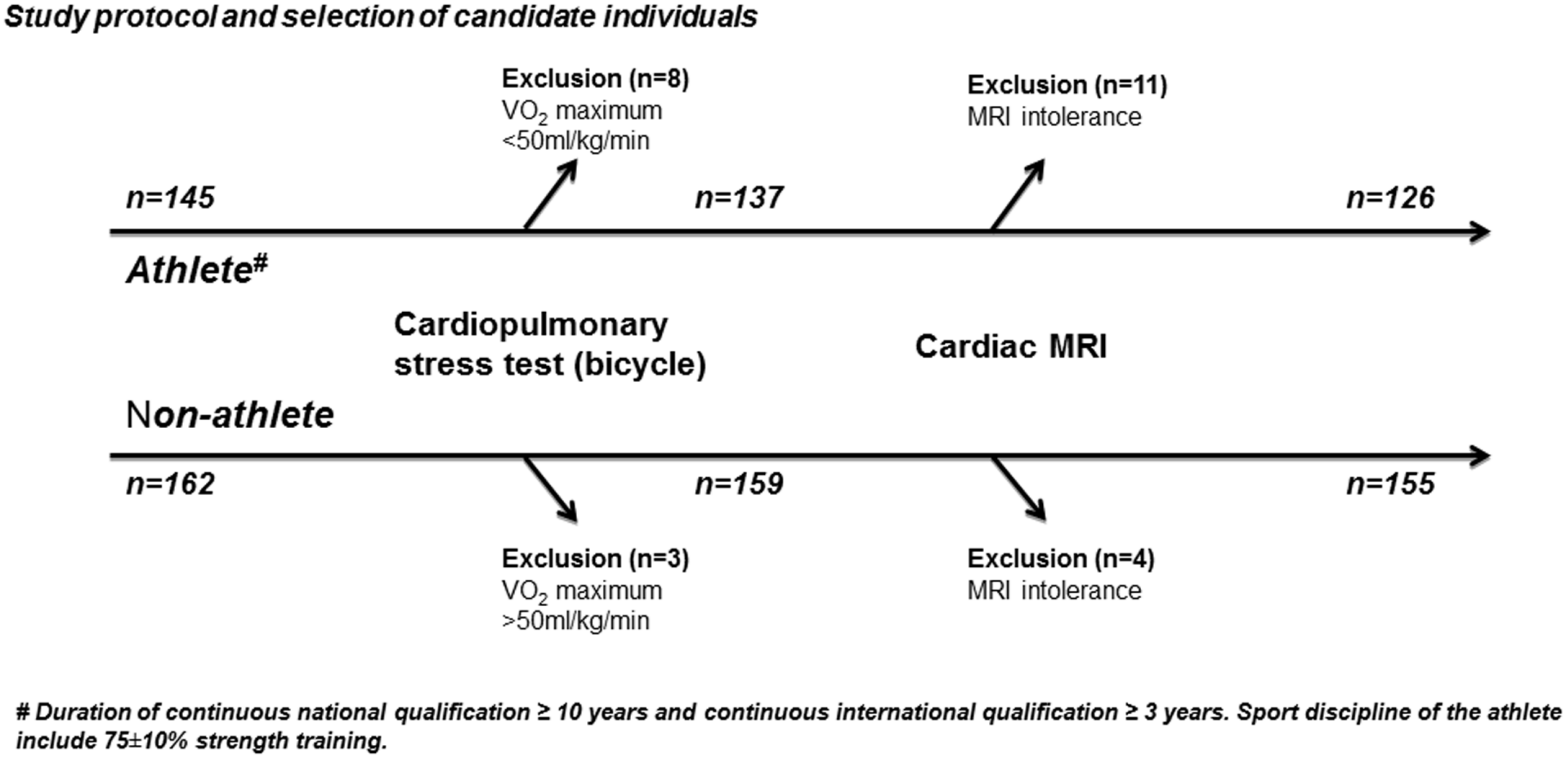
Study protocol and selection of candidate individuals. Top level Hungarian athletes (n = 145) and healthy control individuals were screened. Athletes above and controls under a VO_2_ maximum of 50ml/kg/min were referred to cardiac magnetic resonance (cMRI). Eight athletes were excluded due to low VO_2_ maximum (<50ml/kg/min) and eleven athletes did not complete the cMRI examination due to intolerance. Control individuals with higher than 50ml/kg/min VO_2_ maximum (n = 3) were also excluded and four volunteers could not tolerate cMRI (n = 4).

Hungarian athletes were screened and selected on the basis of event/sport participation, high level qualification and recent international representation. Inclusion criteria consisted of at least 10 years of national and 3 years of international qualifications (world championships and / or Olympic Games). (Fig 1). Athletes with VO_2_ maximum greater than 50ml/kg/min during cardiopulmonary stress test using a bicycle ergometer were referred to cardiac magnetic resonance (cMRI). Eight players were excluded due to low VO_2_ maximum (<50ml/kg/min) and eleven athletes did not complete the cardiac magnetic resonance examination (cMRI) due to intolerance (Fig 1). Sport disciplines with mixed exertion load, including speed, strength and endurance components were selected, therefore water polo players (n = 48), kayakers (n = 21), canoeists (n = 19), rowers (n = 22) and swimmers (n = 16) were involved. Majority of the water polo players were Olympic level athletes (36/48), while in the kayaker group 10/21, in the canoeist group 11/19, in the rower group 12/22 and in the swimmer group 13/16 Olympic level athletes—among them 39 gold medalists—were screened. Training protocol of all examined sportsmen contained mainly strength training. Age and sex matched individuals (n = 162) were screened for the control group. Since lower VO_2_ maximum consumption level (<50ml/kg/min) was part of the inclusion criteria in this group, control individuals with higher than 50ml/kg/min VO_2_ maximum (n = 3) were excluded from the study and four volunteers who could not tolerate cMRI (n = 4) were not able to complete the study protocol.

In this study, all athletes belong to the same ethnic group, are subjected to similar environmental factors including dietary habits, smoking status (only 6/126 athletes were active smokers during the study), duration of elite athletic status and timing (during season) of the examinations.

### Screening protocol

Stress test was performed in both athletes and non-athletes using a bicycle ergometer with an “all-out” protocol. Athletes with a VO_2_ maximum greater than 50ml/kg/min and controls with VO_2_ maximum lower than 50ml/kg/min were referred for cardiac magnetic resonance imaging. Blood samples for DNA isolation were collected at the first visit. Athletes were all tested and measured during their competitive season period in two consecutive years. Participation, including cardiac magnetic resonance imaging and blood sampling for DNA extraction was voluntary and part of a prospective athletic screening program. Written informed consent was collected from all participants and approval of the Hungarian Scientific Council National Ethics Committee for Scientific Research (ETT-TUKEB 13687-1/2011) was provided before data and samples were used in this study. The Hungarian Scientific Council National Ethics Committee for Scientific Research (ETT-TUKEB) is a supreme authority of the Semmelweis University Ethics Committee, which received and approved the national level decision.

### Cardiopulmonary stress test

A continuous ramp test to exhaustion was performed on an electromagnetically braked bicycle ergometer (Ergoline Ergometrics 900, Bitz, Germany). The initial exercise load was 50 W and increased in a linear ramp pattern with 25 W every 60 seconds. Athletes and non-athlete individuals were asked to continuously pedal until exhaustion, maintaining constant revolutions-per-minute at 40–50 rpm. Gas exchange parameters and ventilatory variables were recorded breath-by-breath (PowerCube, Ganshorn Medizin, Niederlauer, Germany). Vital parameters and blood lactate levels were measured before and after the stress test. Rating of perceived exertion (BORG, scale 6–20) was collected and exhaustion was defined by completing the BORG scale.

### Cardiac magnetic resonance imaging (cMRI)

Cardiac MRI scans were performed on a Philips Achieva 1.5T magnet. (Philips Healthcare, Eindhoven, The Nederlands). The imaging instrument had Dual Nova HP gradients (maximum strength: 33/66 mT/m, slew rate: 180/90 mT/m/ms) and running software version R2.5.3 and recently R2.6.3. (Philips Healthcare, Eindhoven, The Nederlands) Five element cardiac coil was used for signal reception. The MR protocol included retrospectively gated balanced steady-state free precession cine movies in three long axis orientations (two-chamber, four-chamber and left-ventricular outflow tract views) and short axis slices covering both ventricles. Slice thickness was 8mm, while inter-slice gap was set to zero. Each cardiac cycle was divided into 25 to 30 phases. Triggered blood suppressed T2-weighted spectral inversion recovery (T2w-SPIR) sequence was used for edema detection. Delayed enhancement images were recorded in the same views as the cine movies to assess abnormal contrast uptake. While administering the Gadovist contrast (0.125mmol/kg IV) k-t BLAST (Broad-use Linear Acquisition Speed-up Technique) balanced turbo field echo (b-TFE) sequence was used to capture rest perfusion datasets of the three long axis slices. Respiratory motions were corrected using breath-holds in end-expiration. In selected cases coronary origins were depicted using a fat-suppressed 3-dimensional b-TFE sequence utilizing respiratory navigator with prospective motion correction. Medis QMASS MR 7.1 and 7.2 (Medis medical imaging systems bv, Leiden, The Netherlands) were used for evaluation. Endocardial and epicardial contours were traced manually for both the left and the right ventricles and volumetric measures (including papillary muscles), ejection fractions, maximal end-diastolic wall thickness and maximal end-diastolic wall thickness, left ventricular end diastolic volume index ratios were determined[5]. Body height and weight were measured and archived in SI units. Body surface area was calculated using the Mosteller formula based on the subject's weight and height.

### DNA extraction and genotyping

Genomic DNA was isolated from whole peripheral blood with a protease based technique (Flexigene DNA System, Qiagen, Hilden, Germany). Samples (1 ml) were added to a lysis buffer and were thoroughly mixed and centrifuged. After discarding the supernatant, samples were denaturized, DNA was ethanol precipitated and reconstituted in the provided buffer. Samples were stored at -80°C. Estimation of the DNA yield and quality control was done by spectrophotometry and determination of the 260/280 absorption ratio (Nanodrop-2000, Thermo Scientific, Wilmington, USA). Genotyping of The Glu298Asp single nucleotide polymorphism (dbSNP: rs1799983, OMIM: +163729) was done with RT-qPCR (StepOne Plus, Applied Biosystems). Pre-designed primers were provided by Applied Biosystems (kit number: C___3219460_20) and the reaction was performed according to the manufacturer’s protocol. For each run parallel samples with positive controls were used. Genetic analysis was performed blinded to patient data, with the provided software. Results are presented according to the National Heart, Lung, and Blood Institute recommendations on reporting genetic results in research studies [24].

### Statistical analysis

Data are presented as mean ± SD for continuous variables, or n (%) for categorical variables. Comparisons between two groups were performed using Student’s t-test for continuous variables (MR parameters), chi-square test for categorical data (genotype, gender and athletic status). Analysis of variance (ANOVA) indicated that genotype and athletic status may influence right ventricular indices (post-hoc test: Tukey HSD). Linear regression was used to explore whether gender and genotype are independent predictors for changes in right ventricular stroke volume index (RVSVi) and right ventricular mass index (RVMi). Mulivariate analysis was performed on groups based on genotype and athletic status (Aspartate carriers + non-athletes; Non-aspartate carriers + non-athletes; Aspartate carriers + athletes and Non-aspartate carriers + non-athletes). P values less than 0.05 were considered significant. Calculations were performed using the SPSS 22.0 program package (IBM Corporation).

## Results

### Characteristics of athletes and control individuals

Elite athletes, including men (n = 94) and women (n = 32) and Hungarian non-athletic controls (n = 109 men and n = 46 women) were screened (Table 1). Age distribution and gender ratio (25.4% and 29.7% female in athletes and non-athletes, respectively) were not different between the study groups. Height, weight and body mass index were higher in men versus women and in athletes versus non-athletes (Table 1). Body surface area, an important value in the interpretation of cardiac magnetic resonance imaging results, was also higher in athletes when compared with non-athletic controls (Table 1).

**Table 1:** Characteristics of athletes and non-athlete individuals.

	Athletes			Non-athletes		
	*All (n = 126)*	*Men (n = 94)*	*Women (n = 32)*	*All (n = 155)*	*Men (n = 109)*	*Women (n = 46)*
***Age (years)***	25.9±5.5	26.9±5.7	24.9±5.3	27.4±5.2	27.1±5.1	28.0±5.7
***Height (m)***	1.87±0.11 *	1.95±0.04 †	1.77±0.07 ‡	1.76±0.11	1.83±0.07	1.67±0.09
***Weight (kg)***	85.2±15.6 *	97.5±8.3 †	72.3±10.2 ‡	70.5±15.3	81.4±13.6	59.9±7.2
***Body mass index (kg/m*** ^***2***^ ***)***	24.8±2.6	25.8±2.2	23.4±2.5	22.8±4.3	24.1±4.6	21.6±3.9
***Body surface area (m*** ^***2***^ ***)***	2.18±0.28 *	2.38±0.1 †	1.89±0.3 ‡	1.94±0.3	2.08±0.3	1.74±0.2

* *p*<0.01 versus all non-athletes,

^†^ *p*<0.01 versus men non-athletes,

^‡^ *p*<0.01 versus women non-athletes.

Body mass index was different between athletes and non-athletes and between men and women (*p*<0.05). Height, weight, body surface area and body mass index was different between men and women individuals in the athlete and non-athlete groups (*p*<0.05). Age was not different between study groups.

### Cardiac morphology and function in athletes and controls

Left ventricular end diastolic and end systolic volumes and left ventricular myocardial mass, indexed to body surface area (LVEDVi, LVESVi, LVMi, respectively) were significantly higher in athletes than in the non-athletic group, suggestive of eccentric hypertrophy (Table 2). Resting LV ejection fraction (LVEF %) was not different between athletes and non-athletes (58.5±6.3% versus 59.4±4.3%, respectively, NS), but LV stroke volume index (LVSVi) was higher in athletes than in untrained controls (67.6±8.3 versus 54.8±7.8 ml/m^2^, *p*<0.0001). There were significant gender-related differences with higher end-diastolic and end-systolic LV volumes and larger LV mass in men versus women, irrespective of athletic activity (Table 2). Similarly, RV end-diastolic and end-systolic volume indexes (RVEDVi, RVESVi) were higher in athletes compared to non-athletes and again, higher in men than in women (Table 2). Resting RV stroke volume index (RVSVi) and RV mass index (RVMi) were both higher in athletes compared to non-athletes (for RVSVi 68.2±10.2 versus 56.7±7.0 ml/m^2^, p<0.05 and for RVMi 29.9±6.1 versus 24.4±4.3 g/m^2^, p<0.05). Both, RVSVi and RVMi were higher in men than in women. At exhaustion both VO_2_ maximum, (60±7 versus 40±7 ml/kg/min, *p*<0.0001) and minute ventilation (V_E_, 150±15 versus 84±39 l/min, *p*<0.0001) were significantly higher in athletes than in non-athletic controls and within each group, also significantly higher in men than in women.

**Table 2:** Characteristics of athlete and non-athlete men and women.

	Athletes			Non-athletes		
	All	Men	Women	All	Men	Women
**n**	126	94	32	155	109	46
***LVEF (%)***	58.5±6.3	57.8±4.3	60.4±9.7	59.4±4.3	59.5±4.4	58.4±4.2
***LVEDVi (ml/m*** ^***2***^ ***)***	116.2±17.4 *	121.4±14.9 ‡	102.4±16.2	92.9±12.9	97.3±11.1 ‡	84.4±13.2
***LVESVi (ml/m*** ^***2***^ ***)***	48.8±11.2 *	51.4±9.6 ‡	41.9±12.1	37.8±7.6	39.5±7.3 ‡	35.4±8.0
***LVMi (g/m*** ^***2***^ ***)***	81.1±19.6 *	88.1±15.9 ‡	62.4±16.0	61.3±13.9	68.1±10.4 ‡	47.0±10.1
***LVSVi (ml/m*** ^***2***^ ***)***	67.6±8.3 *	69.8±8.1 ‡	61.6±5.6	54.8±7.8	57.8±6.8 ‡	49.0±6.8
***RVEF (%)***	57.9±6.2	57.1±4.0	60.2±9.9	58.5±4.8	58.2±4.9	58.2±4.4
***RVEDVi (ml/m*** ^***2***^ ***)***	121.5±19.6 *	127.8±17.4 ‡	106.0±17.3	95.5±15.2	100.7±14.1 ‡	85.7±13.4
***RVESVi (ml/m*** ^***2***^ ***)***	53.2±11.3 *	55.8±10.9 ‡	46.6±9.9	39.8±9.6	42.6±9.9 ‡	35.3±7.3
***RVSVi (ml/m*** ^***2***^ ***)***	68.2±10.2 *	70.7±9.5 ‡	61.5±9.2	56.7±7.0	58.3±6.4 ‡	52.4±7.7
***RVMi (g/m*** ^***2***^ ***)***	29.9±6.1 *	32.5±4.6 ‡	23.5±5.4	24.4±4.3	25.3±3.5 ‡	20.1±3.0

n—number of individuals; LVEF—left ventricular ejection fraction; LVEDVi—left ventricular end-diastolic volume index; LVESVi—left ventricular end-systolic ventricular index; LVMi—left ventricular mass index; LVSVi—left ventricular stroke volume index; RVEF—right ventricular ejection fraction; RVEDVi—right ventricular end-diastolic volume index; RVESVi—right ventricular end-systolic volume index; RVSVi—right ventricular stroke volume index; RVMi—right ventricular mass index.

* *p*<0.01 versus all non-athletes,

^‡^ *p*<0.01 versus women.

Age was not different between study groups.

### Genotype distribution

Allelic distributions in athletes and controls were similar with aminor allelic frequency of 0.27 in athletes vs. 0.26 in controls. In 64 athletes the Glu homozygous genotype, in 56 the heterozygous while in 6 out of 126 athletes the Asp homozygous genotype was found. The genotype distribution was similar in the non-athletic control group (84/ G/G, 62/ G/A, and 9/ A/A, chi-square = 0.62; *p* = 0.73, Table 3). Therefore the investigated SNP did not correlate with athletic status (*p* = 0.59, with OR (CI 95) of 0.8 (0.5–1.4). The proportion of the G/G genotype varied between 49 and 57% in both genders in both groups. Overall, genotype distributions were in Hardy-Weinberg equilibrium for the SNP in the population.

**Table 3:** Nitric oxide synthase 3 gene 298 Glu/Asp genotype distribution in athletes and in non-athlete controls.

	Athlete(VO_2_ max >50ml/kg/min)		Non-athlete(VO_2_ max <50ml/kg/min)	
	Men	Women	Men	Women
**GG (Glu/Glu)**	46 (49%)	18 (56%)	58 (53%)	26 (57%)
**GT (Glu/Asp)**	43 (46%)	13 (41%)	45 (41%)	17 (37%)
**TT (Asp/Asp)**	5 (5%)	1 (3%)	6 (6%)	3 (6%)

Number of individuals with different genotypes in the different groups. Percentage in brackets represents the allelic frequency within the subgroup.

### Association of cardiac structure and function with the *NOS3* 298 genotype

Resting RV stroke volume index (RVSVi) and RV mass index (RVMi) were both higher in Aspartate carriers (athletes and non-athletes, n = 133) compared to Glutamate homozygous participants (athletes and non-athletes, n = 148), for RVSVi 60±9 versus 62±12 ml/m^2^ (p = 0.047, t-test) and for RVMi 26±6 versus 27±6 g/m^2^ (p = 0.019, t-test).

Athletes carrying the Asp allelic variant had higher right ventricular mass index, than those homozygous for the Glu allelic variant (31.7±5.5 versus 27.4±6.0 g/m^2^, p<0.01, Tables 4 and 5). RVMi represents right ventricular hypertrophy and was associated with higher right ventricular stroke volume index (RVSVi) in the Asp allele carrier versus the Glu homozygous athlete group (71.1±9.6 versus 64.3±9.8 ml/m^2^, p<0.001, Tables 4 and 5). In non-athletic individuals no association was observed between genotype and right ventricular function and mass. Similarly, left ventricular function and anatomy was not associated with the genotype in any of the study groups (Table 4). Oxygen consumption at exhaustion (VO_2_ maximum) was not different between the Asp allelic variant carriers and the Glu homozygous individuals, irrespective of the athletic status (in athletes 61±8 versus 58±6 ml/kg/min and in non-athletes 40±7 versus 39±7 ml/kg/min, respectively, NS for both). There was, however, a trend towards higher maximal minute ventilation in Asp allelic variant carrier versus Glu homozygous athletes (152±13 versus 146±11 l/min, *p* = 0.08). The genotype was not associated with ventilation in non-athletes.

**Table 4:** Characteristics of different genotypes within athletes and non-athletes (irrespective of their gender).

	Athletes (VO2 max >50ml/kg/min)		Non-athletes (VO2 max >50ml/kg/min)		
	Glu/Glu	Glu/Asp + Asp/Asp	Glu/Glu	Glu/Asp+ Asp/Asp	p
**n**	64	62	84	71	-
***LVEF (%)***	58.3±8.1	58.7±4.5	59.1±2.4	60.0±6.7	0.688
***LVEDVi (ml/m*** ^***2***^ ***)***	115.1±20.5 *	117.0±14.9 *	92.1±14.6	94.4±9.3	<0.001
***LVESVi (ml/m*** ^***2***^ ***)***	49.2±13.3 *	48.5±9.3 *	37.8±7.8	37.9±7.6	<0.001
***LVMi (g/m*** ^***2)***^	77.9±23.1 *	83.5±16.2 *	60.3±15.3	63.2±11.2	<0.001
***LVSVi (ml/m*** ^***2***^ ***)***	66.4±7.6 *	68.5±8.7 *	54.1±7.6	56.1±8.2	<0.001
***RVEF (%)***	57.7±8.1	58.1±4.4	58.3±4.1	58.4±5.8	0.427
***RVEDVi (ml/m*** ^***2***^ ***)***	117.8±19.9 *	124.4±19.1 *	95.6±17.6	95.4±9.8	<0.001
***RVESVi (ml/m*** ^***2***^ ***)***	53.0±10.5 *	53.4±12.1 *	40.4±11.0	38.7±6.5	<0.001
***RVSVi (ml/m*** ^***2***^ ***)***	64.3±9.8 # *	71.1±9.6 *	56.6±6.7	56.8±7.8	<0.001
***RVMi (g/m*** ^***2***^ ***)***	27.4±6.0 # *	31.7±5.5 *	25.3±4.7	23.3±3.4	<0.001

n—number of individuals; LVEF—left ventricular ejection fraction; LVEDVi—left ventricular end-diastolic volume index; LVESVi—left ventricular end-systolic ventricular index; LVMi—left ventricular mass index; LVSVi—left ventricular stroke volume index; RVEF—right ventricular ejection fraction; RVEDVi—right ventricular end-diastolic volume index; RVESVi—right ventricular end-systolic volume index; RVSVi—right ventricular stroke volume index; RVMi—right ventricular mass index

Analysis of variance showed significant differences among the inspected groups: athletes and non-athletes with and without the Aspartate allele. Post hoc tests revealed a significant influence of the genotype on resting RVSVi and RVMi in athletes,

^#^ p<0.001 vs Asp carriers within the athlete group.

Athletic status had significant influence on all parameters with the exception of LVEF and RVEF,

* p<0.001 vs non-athletes irrespective of genotype.

**Table 5:** Characteristics of male and female athletes and athletes with, or without the Asp allele.

		Resting right ventricular stroke volume index (RVSVi, ml/m2)	Right ventricular mass index (RVMi g/m2)
**Gender**	**Men**	70.7±9.5 ‡	32.5±4.6 ‡
	**Women**	61.5±9.2	23.5±5.4
***NOS3***	**Glu/Glu**	64.3±9.8	27.4±6.0
	**Asp carrier**	71.1±9.6 #	31.7±5.5 #

In this age matched population of elite athletes with a similar social and ethnic background linear regression analysis revealed that both male gender and the presence of the Asp allele are independent predictors for higher resting right ventricular stroke volume index (RVSVi) and right ventricular mass index (RVMi) values.

^‡^ p<0.001 vs women,

^#^ p<0.001 vs Aspartate carriers.

## Discussion

In the present study, association between sport-related right ventricular adaptation and the Glu298Asp genetic variant of the endothelial nitric oxide synthase 3 gene was examined in elite athletes. The *NOS3* genotype and its relation with athletic performance and physiologic adaptation in selected elite athletes have already been described, however we are first to report that physical preconditioning evokes genotype-influenced right ventricular adaptation. [21,22,25]

Consistent with previous findings, in our study, no difference in genotype distribution was found between elite athletes and control individuals [23]. According to the dbSNP database Minor allele frequency was lower than in the HapMap-CEU population (0.27 vs. 0.34) and comparable with the largest population available (PA159018372, 0.27 vs. 0.29).

Although the adaptation of the left ventricle and its possible functional consequences on athletic performance represent a major focus of sport physiology research, there is increasing evidence that right ventricular adaptation may be a better marker of athletic performance [1,3,26]. There is a near linear correlation between exercise intensity and pulmonary artery pressures, which results in a disproportionate increase in right, compared to the left ventricular afterload [6]. Association between strenuous high intensity exercise and a disproportionate enlargement of the right ventricle was observed previously. [4,27]. Beside chamber dilation athletic adaptation goes together with decreased resting RV ejection fraction and ventricular-arterial coupling alterations which, in turn, may be an early sign for contractile impairment [28]. In addition right ventricular mass and volume were also independent markers of self-reported physical activity in a recent community-based trial [26]. These changes in the RV structure represent physiologic athletic adaptations, however, extensive remodeling may also predict pathologic conditions. Distinguishing physiologic- from pathologic adaptation remains an important and challenging task [29,30]. Therefore, a careful examination of the right ventricle in highly trained individuals is therefore critical, although it is not part of the current pre-participation screening protocols [31].

Because of its anti-hypertrophic myocardial effects nitric oxide (NO) could affect athletic performance via several mechanisms, including improved coupling of cardiac oxygen consumption to physical performance, enhanced LV relaxation and decreased LV end-diastolic pressure, increased NO-dependent myocardial Ca^2+^ influx and contractile force [11,32–34]. The primary source of NO is NOS3 in the cardiovascular system. Therefore, Glu to Asp amino acid substitution at codon 298 in the NOS3 enzyme and reduced NO production may result in altered biological effects, including impaired vasodilation and increased myocardial hypertrophy.

In our study, however, no connection was found between the *NOS3* genotype and left ventricular parameters neither in athletes, nor in untrained-individuals. Interestingly, without a significant effect on maximal oxygen uptake, there was a trend towards higher minute ventilation at peak exercise in Asp allelic carrier compared to Glu homozygous athletes. This phenomenon was not observed in non-athletes.

Bench-side and clinical data support the theory that the hypoxia-induced pulmonary vasoconstriction may be further enhanced by the increased cleavage of the Aspartate containing *NOS3* protein [6,13]. Under excessive chronic load this could hasten the depletion of the marginally sufficient right ventricular contractile reserves, which was shown to lead to a disproportionate and exaggerated right ventricular remodeling in both animal- and clinical studies [6,35,36]. As sustained right ventricular fatigue and injury may result in pathologic fibrous remodeling, the clinical significance of our findings could lie with their potential influence on the development of RV cardiomyopathy [7,37].

The influence of the *NOS3* Glu298Asp polymorphism on enzymatic cleavage and thus NO availability has been shown to reduce treatment efficacy in heart failure patients. In a genetic sub-study of the African-American Heart Failure Trial (A-Heft) Glu298Glu genotype predominance was associated with the positive impact of NO donors (isosorbide dinitrates and hydralazine) on heart failure survival [12,13].

The *NOS3* Glu298Asp polymorphism has also been associated with cardiac adaptation to long-term athletic performance. This association included the cardiovascular adaptation of untrained individuals to long term endurance training with a blunted responsiveness of submaximal exercise, diastolic blood pressure and rate pressure product in previously sedentary Asp homozygous subjects. Higher stroke volume and lower heart rate during sub-maximal exercise was reported in postmenopausal women carrying the Asp allelic variant [21,25]. The *NOS3* Glu298Asp variant was also associated with the actual contest performance in elite ultra-endurance athletes [22].

In elite athletes we found larger right ventricular myocardial mass and increased RV stroke volume (both indexed to body surface area) in Asp allele carriers versus Glu homozygous individuals. The higher stroke volume observed in the Asp carrier athletes was associated with a non-significant trend of larger end diastolic volumes compared to the Glu homozygous athletes.

These associations could be the consequences of the increased afterload and adverse pulmonary remodeling brought forth by reduced NO bioavailability. Knockout murine experiments highlighted the fact that albeit all three isoforms are present in the lung, NOS3-derived NO is the major regulator of pulmonary vascular tone. Loss of NOS3 function results in a markedly enhanced hypoxic pulmonary vasoconstriction, significantly elevates right ventricular pressure and induces airway hyper-responsiveness [38,39]. In addition, even the endothelial response to ß-adrenergic stimuli could be compromised and thus protection from sympathetic hyperactivity could be hampered [40]. During the last decade candidate gene approach and analysis of single genetic variants became overshadowed by systems biology, gene-environmental and gene-gene interaction studies. In the present study group however strict inclusion criteria make the analysis of correlations with a single genetic variant more plausible.

### Limitations

Cross sectional study design limits insight on the interaction of the *NOS3* Glu298Asp polymorphism with athletic remodeling. As this study was focused on a highly selected group of athletes, individuals from several sports disciplines—although with very similar training regimes—had to be screened to reach an acceptable number of participants. Given our single gene candidate approach, other polymorphisms in linkage disequilibrium with the Glu298Asp polymorphism may be the true cause of this association.

## Conclusion

In this study cardiac function and structure of elite athletes was determined and a previously unknown correlation was found between load-dependent right ventricular adaptation and a candidate genetic variant, the *NOS3* Glu298Asp polymorphism. Our study supplements evidence in the literature showing another aspect of NOS3 genotype influence on cardiac adaptation. Whether this has a positive or negative effect on the individuals’ long term cardiovascular health and athletic performance has yet to be determined. Combined use of non-invasive cutting edge imaging modalities and genotyping tools may help to track the extent of influence both during training and late life deconditioning.

Given the high frequency of the nitric oxide synthase 3 gene 298 Glu/Asp polymorphism this could have substantial impact if there was more evidence related to its functional and long-term implications.
